# Insights into high-altitude adaptation and meat quality regulation by gastrointestinal metabolites in Tibetan and black pigs

**DOI:** 10.3389/fvets.2025.1569196

**Published:** 2025-03-26

**Authors:** Xue Bai, Zhiying Huang, Helin Tan, Yiren Gu, Xun Wang, Long Jin, Peng Shang, Keren Long, Diyan Li, Mingzhou Li

**Affiliations:** ^1^College of Animal and Veterinary Sciences, Southwest Minzu University, Chengdu, China; ^2^State Key Laboratory of Swine and Poultry Breeding Industry, College of Animal Science and Technology, Sichuan Agricultural University, Chengdu, China; ^3^Animal Science College, Xizang Agriculture and Animal Husbandry University, Linzhi, China

**Keywords:** Tibetan pig, black pig, metabolome, altitude, muscle

## Abstract

**Introduction:**

Tibetan pigs, native to the Qinghai-Tibet Plateau, have adapted over millennia to extreme conditions such as low oxygen, harsh cold, and high UV radiation, impacting their muscle characteristics and digestive tract microbiota. The quality of pork from Tibetan pigs (TP) and black pigs (BP) is influenced by various factors, including genetics, diet, and environmental adaptation. However, the specific influence of digestive tract microbiota metabolites on muscle traits remains poorly understood. Our goal was to correlate omic variations with meat quality traits and identify potential biomarkers predictive of superior meat quality, elucidate the regulatory effects of digestive tract microbial metabolites on Tibetan pig muscle characteristics, and reveal the genetic and nutritional mechanisms that promote adaptation to extreme environmental conditions.

**Methods:**

This analysis encompassed metabolomic profiling of the entire digestive tract-including the stomach, jejunum, cecum, colon, and rectum-as well as histological, amino acid, fatty acid composition, and transcriptomic assessments of the longissimus dorsi muscle tissues to investigate how digestive tract microbial metabolites influence muscle adaptation to high altitudes.

**Results:**

Analyses revealed that Tibetan pig muscles contain smaller, more oxidative fibers enriched with flavor-enhancing amino acids. This was accompanied by a more favorable n-6/n-3 fatty acid ratio. Distinct patterns of microbial metabolites were observed in the digestive tract, influencing protein digestion and purine metabolism, and correlating with muscle glycine levels. Transcriptomic data showed varied gene expression in metabolic pathways related to salivary and pancreatic secretion, as well as carbohydrate and fatty acid metabolism. Integrated multi-omics approaches linked stomach metabolism, particularly through bile secretion pathways influenced by acetylcholine, to muscle functionality, highlighting the important role played by the ATP1B4 gene in enabling muscle physiology in Tibetan pigs.

**Discussion:**

This study highlights the importance of targeted dietary interventions in improving meat quality for specific pig breeds. It also provides a theoretical foundation for precision agriculture strategies aimed at enhancing the meat quality of both TP and BP pigs.

## 1 Introduction

The Tibetan pig, a highland breed native to the Qinghai-Tibetan plateau in China (1, 2), has adapted to high altitudes and low-oxygen environments, resulting in distinctive genetic and physiological traits compared to conventional domestic pigs (3, 4). Raised free-range on natural pastures, Tibetan pigs produce pork known for unique qualities and high nutrient content (5). Although Tibetan pigs have slower growth rates, they accumulate more fat, an adaptation to the cold climate and energy demands of their environment (6, 7). Comparative studies reveal that Tibetan pork differs significantly in taste and flavor from leaner commercial breeds, such as Yorkshire, due to its distinctive aroma compounds, fatty acid composition, and amino acid profile, which enhance its palatability (8). To combine these favorable traits, we crossed Tibetan pigs with Duroc pigs, creating hybrid Sichuan black pigs that exhibit robust adaptability, high disease resistance, rich meat flavor, and rapid growth. Black pigs, when compared to commercial white breeds, display superior meat quality traits, including enhanced marbling, tenderness, and flavor (9, 10). Due to these qualities, black pork is highly valued by consumers, commanding prices two to three times higher than standard white pork and offering considerable economic benefits.

The gut serves as the primary site for nutrient digestion and absorption (11). The gut microbiota, a dense community of microorganisms, acts as a bridge between the animal and its nutritional environment (12). Essential for nutrient processing, the gut microbiome plays a key role in the host's physiological, nutritional, and immune functions (13, 14). It communicates with the host through various metabolic products (15, 16). Increased rumen microbiota and higher levels of short-chain fatty acids can influence important metabolic products such as inosine, riboflavin, AMP, ADP, and L-glutamate, which regulate purine metabolism and impact the brightness and tenderness of lumborum muscle (17). Dietary interventions in sheep have been shown to modify muscle metabolism of amino acids, lipids, and carbohydrates (18). These changes in muscle metabolism are accompanied by shifts in the composition of the gut microbiome, which actively participates in the metabolic incorporation of amino acids and fatty acids. Collectively, these factors affect the amino acid composition, fatty acid content, pH, as well as the color, tenderness, and water-holding capacity of the meat (19). These findings underscore the critical role of the intestinal microbiota in regulating digestion and its profound impact on meat quality through complex metabolic and dietary interactions.

Muscle metabolites play a crucial role in the physiological dynamics of muscle tissue and ultimately determine meat quality. Extensive research has investigated metabolic signatures as potential biomarkers for assessing meat quality by analyzing its chemical and metabolomic landscape (20–22). Disruptions in gut microbial populations can alter muscle metabolites, which in turn influence meat quality (23). As a result, dietary optimization, feed management strategies, or the inclusion of probiotics can modify muscle metabolism and the associated phenotypic traits. However, few studies have explored the intestinal metabolomics and meat quality characteristics of Tibetan and black pigs. This study uses metabolomics to examine fecal metabolome variations across different intestinal sections in Tibetan and black pigs, linking these findings to meat quality. By doing so, we aim to better understand how breed influences intestinal metabolism. This research offers a novel approach to investigating the impact of breed on intestinal metabolic profiles and provides a basis for regulating the meat quality of Tibetan and black pigs through feeding and management strategies.

## 2 Materials and methodology

### 2.1 Animal management and sample collection

In this study, we used six Sichuan local Gaojin black pigs (Suining, Sichuan, China, 30°53′N, 105°59′E, altitude 461 m) and six Tibetan pigs (Daocheng, Sichuan, China, 28°26′N, 99°86′E, altitude 3,750 m). After 300 days of stable housing, we transported all animals to a nearby commercial slaughterhouse. Following standard animal welfare protocols, the pigs were fasted for 24 h and water-deprived for 2 h before slaughter. We then harvested the thoracic segment of the longissimus dorsi (LD) muscle, carefully removing any superficial fat and fascia. To collect intestinal samples, we separated the different intestinal sections and extracted contents from the stomach, jejunum, cecum, colon, and rectum. Immediately after collection, all samples were flash-frozen in liquid nitrogen, transferred to dry ice for temporary storage, and then transported to the laboratory for storage at −80°C. We obtained six biological replicates and three technical replicates for each breed.

### 2.2 Fluorescence homologous double-label staining of muscle paraffin sections

Fresh tissue samples were dissected and immediately placed in an appropriate fixative for over 24 h at room temperature to stabilize them for transport. After dehydration, paraffin embedding, and sectioning, we applied BSA (10% rabbit serum) to block the sections for 30 min. Following removal of the blocking solution, we added the first primary antibody, placed the sections flat in a humidified chamber, and incubated them overnight at 4°C.

The sections were then washed with PBS (pH 7.4), gently spun, treated with TSA, and incubated for 10 min in the dark at room temperature. After incubation, we rinsed the slides in TBST on a destaining shaker. For antigen retrieval, we placed the sections in a repair box filled with antigen retrieval buffer and heated them in a microwave. We then applied the second primary antibody, incubated overnight at 4°C, and washed the sections. Following this, we added the appropriate fluorescent secondary antibody and incubated for 50 min at room temperature in the dark. After washing, we applied DAPI dye solution for 10 min at room temperature in the dark. We then added autofluorescence quencher B solution, incubated for 5 min, and rinsed with running water for 10 min. Finally, we mounted the slides with an anti-fluorescence quenching medium. We captured images of the sections using a digital pathology slide scanner (3DHISTECH, Pannoramic MIDI) and measured muscle fiber cross-sectional area. Additionally, we assessed different muscle fiber types and quantified muscle fiber numbers using Image-Pro Plus software.

### 2.3 Targeted metabolomic analysis of LD

We ground the freeze-dried LD sample and placed 0.1 g into an ampoule. We added 10 mL of 6 mol/L hydrochloric acid for hydrolysis, froze the sample in liquid nitrogen for 1–2 min, sealed the ampoule, and hydrolyzed it in a thermostat at 110 ± 1°C for 22 h. After cooling to room temperature, we filtered the hydrolysate, adjusted the volume to 50 mL in a volumetric flask, and mixed thoroughly. We then transferred 1.0 mL of the filtrate to a 15 mL test tube and dried it under reduced pressure at 45°C using a parallel evaporator. After complete drying, we dissolved the residue in 1 mL of water, dried it again under reduced pressure, and then evaporated it to dryness. We reconstituted the dried material in 1.0 mL of pH 2.2 sodium citrate buffer, mixed well, and filtered it through a 0.22 μm membrane for analysis. Determination of fatty acid composition. For fatty acid composition analysis, we weighed 0.3 g of sample into a 6 mL centrifuge tube, added 3 mL of a methanol (1:2) solution, and agitated it on a shaker for 1 h. After filtering through quantitative filter paper, we added 3 mL of distilled water, centrifuged the sample at 3,000 rpm for 5 min, removed the supernatant, and dried the lower layer in a water bath under reduced pressure at 40°C. We then dissolved the oil residue in 1 mL of chromatographically pure n-hexane, added 1 mL of 0.4 mol/L KOH-methanol solution, and allowed it to stand for 30 min for methyl esterification. After layering, we added 2 mL of deionized water, extracted the upper solution, and analyzed it using gas chromatography.

### 2.4 RNA extraction and transcriptome sequencing

We isolated and purified total RNA from LD tissue samples using a total RNA extraction kit. We assessed RNA purity with a Nanophotometer Spectrophotometer (IMPLEN, CA, USA) and evaluated RNA integrity using the Agilent Bioanalyzer 2100 system with the RNA Nano 6000 Assay Kit (Agilent Technologies, CA, USA). We then generated sequencing libraries with the NEBNext Ultra™ RNA Library Prep Kit for Illumina (NEB, USA) and performed sequencing on the Illumina NovaSeq 6000 platform. To process the raw sequencing data, we used SOAPnuke with parameters (-n 0.001, -l 20, -q 0.4, –adaMR 0.25, –polyX 50, –minReadLen 150) to filter out contaminants, adapters, and low-quality reads. We then mapped the cleaned data to the pig reference genome (Sscrofa11.1) using STAR software (v2.7.6a) (24). For gene expression quantification, we used Kallisto (v0.44.0) (25), calculating transcript levels in transcripts per kilobase million (TPM). We identified differentially expressed genes with DESeq2 (v1.20) (26), designating genes with a corrected *P*-value < 0.05 and a fold change >2 or < 0.5 as significantly differentially expressed. Next, we applied t-SNE analysis to the TPM data using the “factoextra” package (v1.0.7) to visualize sample clustering and generated a correlation heatmap to show Spearman correlation coefficients for sample pairs. Finally, we used the clusterProfiler package (v4.9.2) (27) for functional annotation, including Gene Ontology (GO) term enrichment and Kyoto Encyclopedia of Genes and Genomes (KEGG) pathway mapping.

### 2.5 Untargeted metabolomic analysis

After thawing the gastrointestinal content sample at 4°C, 25 mg was weighed and transferred to a 1.5 mL Eppendorf tube. Next, 800 μL of extraction solution (methanol: acetonitrile: water = 2:2:1, v: v, pre-cooled to −20°C) and 10 μL of internal standard were added. Two small steel beads were also added, and the sample was ground using a tissue grinder at 50 Hz for 5 min. It was then ultrasonicated in a 4°C water bath for 10 min and stored at −20°C for 1 h. Afterward, the sample was centrifuged at 25,000 g for 15 min at 4°C From the resulting supernatant, 600 μL was collected, dried using a vacuum concentrator, and reconstituted with 600 μL of reconstitution solution (methanol: H_2_O = 1:9, v:v). The sample was vortexed for 1 min, ultrasonicated again in a 4°C water bath for 10 min, and centrifuged at 25,000 g for 15 min at 4°C. The supernatant was carefully transferred into a sample vial. To assess the repeatability and stability of the LC-MS analysis, 50 μL of the supernatant from each sample was pooled to prepare QC samples.

We separated and detected metabolites using a Waters 2777C UPLC (Waters, USA) coupled with a Q Exactive HF high-resolution mass spectrometer (Thermo Fisher Scientific, USA). We employed a BEH C18 column (1.7 μm, 2.1 × 100 mm, Waters, USA) for chromatographic separation. For positive ion mode, the mobile phase consisted of an aqueous solution with 0.1% formic acid (Liquid A) and methanol containing 0.1% formic acid (Liquid B). In negative ion mode, the mobile phase included an aqueous solution with 10 mM ammonium formate (Liquid A) and 0.1% formic acid in 95% methanol (Solution B). The Q Exactive HF mass spectrometer (Thermo Fisher Scientific, USA) was used to acquire both primary and secondary mass spectrometry data. The mass range for scanning was m/z 70–1050, with a primary resolution of 120,000, an automatic gain control (AGC) target of 3e6, and a maximum injection time (IT) of 100 ms. We processed the raw mass spectrometry data using Compound Discoverer 3.3 (Thermo Fisher Scientific, USA) software. Data were analyzed in conjunction with the BGI Metabolome Database (BMDB), mzCloud, and ChemSpider online databases.

### 2.6 Statistical analysis

This study used multivariate statistical analysis methods for metabolomics data analysis. In the R language environment, principal component analysis (PCA) and partial least squares discriminant analysis (OPLS-DA) were first used for data dimensionality reduction and pattern recognition. The OPLS-DA model was used to extract the feature variable importance index (VIP), and metabolite screening was achieved by combining hypothesis testing and differential fold analysis: the screening threshold for differential metabolites was set as VIP score higher than 1.0, *t*-test corrected *P*-value less than 0.05, and differential fold FC> 2 or < 0.5. The ggplot2 package was used to construct a multidimensional volcano plot, integrating the VIP score and log2-transformed differential fold significance value. The cluster heat map of metabolite expression patterns was implemented using the Pheatmap package, and the data preprocessing was standardized by Z-score. Metabolic pathway enrichment analysis was based on the KEGG database. The metabolic pathway significance criterion was: the pathway enrichment index obtained by Fisher's exact test had a *P*-value lower than 0.05, and the results were visualized by bubble charts. The Pearson correlation analysis between metabolites and muscle indicators was performed by calculating the correlation coefficient matrix using the cor function, performing statistical tests using cor.mtest, and visualizing using the corrplot package.

## 3 Results

### 3.1 Comparative histological analysis of LD muscle between TP and BP

**Figure 1:**
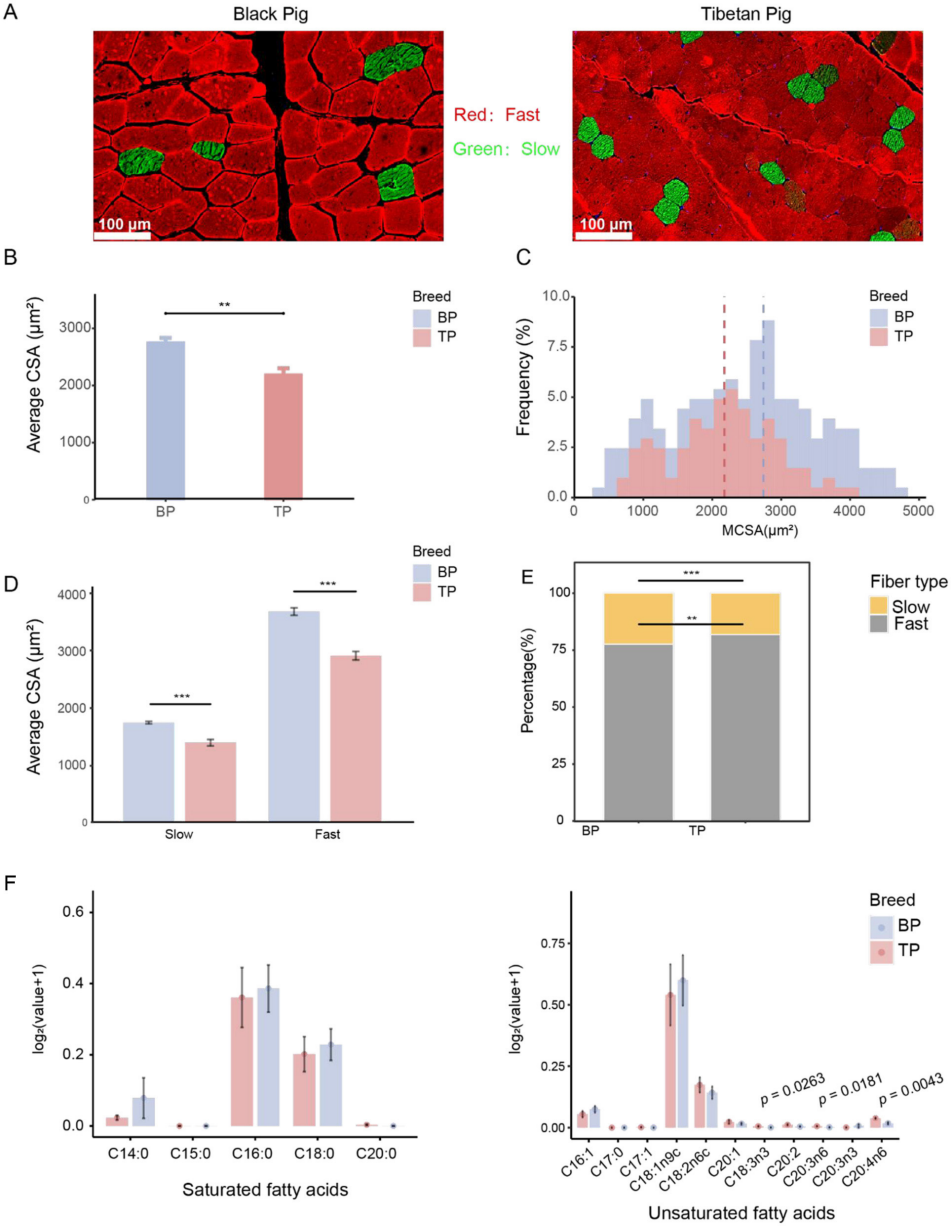
Comparative analysis of between LD muscle fibers of Tibetan pigs and Black pigs. **(A)** Representative images of muscle fiber type staining showing slow-twitch muscle fibers (type I, green) and fast-twitch muscle fibers (type II, red); scale bar represents 100 μm. **(B)** Quantified average muscle fiber area (CSA) of LD. **(C)** Distribution frequency of cross-sectional area (CSA, μm^2^) of LD muscle fibers. **(D)** CSA analysis by fiber type. **(E)** Percentage of muscle fiber type number. **(F)** Histogram of fatty acid content in Tibetan pigs and black pigs, with the ordinate being log2 (fatty acid content g/100g+1). *n* = 6 per group. Significant differences are indicated by *: **p < 0.01, ***p < 0.001.

Understanding the muscle composition of different pig breeds is crucial for developing scientific breeding strategies, optimizing nutritional plans, and enhancing meat production efficiency. This study conducted a comparative histological analysis of the LD muscle between Tibetan pigs and Black pigs (Figure 1A). The results revealed that the average cross-sectional area (CSA) of muscle fibers in the Black pigs' LD muscle was significantly larger than that in Tibetan pigs (BP: 2741.62 ± 217.56 μm^2^, TP: 2175.67 ± 292.12 μm^2^; *p* < 0.01; Figure 1B), and the proportion of small-area muscle fibers was notably higher in Tibetan pigs (Figure 1C). To further explore the potential effects of the high-altitude environment on fiber types, this research delved into the specific changes in muscle fibers. The findings indicated that both the fast-twitch and slow-twitch fibers' CSA in Tibetan pigs were significantly smaller than those in Black pigs, with a higher proportion of fast-twitch fibers in Tibetan pigs (Figures 1D, E, Supplementary Table S1). In summary, the LD muscle of Tibetan pigs exhibited characteristics of reduced muscle fiber area, increased proportion of small-area fibers, and a higher proportion of fast-twitch fibers.

### 3.2 Comparative analysis of amino acid and fatty acid profile in LD muscles in Tibetan and black pigs

This study analyzed the amino acid and fatty acid compositions of the LD muscle in Tibetan and black pigs. The results revealed increase in the total amino acid content in the LD of black pigs (Table 1, Supplementary Table S2). Essential amino acids (EAAs) are a key indicator of meat's nutritional value. The EAA content in the LD of black pigs (9.62 ± 0.498 g/100 g) was higher than that in Tibetan pigs (8.74 ± 0.501 g/100 g). Additionally, the levels of flavor amino acids, including aspartic acid, alanine, arginine, glutamic acid, and glycine, were higher in black pigs (11.46 ± 0.631 g/100 g) than in Tibetan pigs (10.61 ± 0.51 g/100 g). These findings suggest that black pigs have a higher amino acid content, particularly essential and flavor amino acids, which enhance the nutritional value and flavor of pork.

**Table 1:** Amino acid content of long muscle fibers of Tibetan pigs and black pigs.

**Amino acids**	**TP**	**BP**	***p*-value**
	**Mean** ±**SEM (*****n*** = **6)**	**Mean** ±**SEM (*****n*** = **6)**	
Glu	3.13 ± 0.22	3.31 ± 0.29	0.255
Asp	1.93 ± 0.1	2.02 ± 0.16	0.257
Ala	1.11 ± 0.06	1.19 ± 0.1	0.121
Ser	0.78 ± 0.05	0.82 ± 0.09	0.394
Pro	0.70 ± 0.05	0.74 ± 0.12	0.538
Gly	0.95 ± 0.03	0.89 ± 0.05^*^	0.033
Lys	1.85 ± 0.11	1.93 ± 0.16	0.338
Phe	0.80 ± 0.03	0.86 ± 0.06	0.064
Val	0.99 ± 0.05	1.06 ± 0.09	0.116
Leu	1.64 ± 0.1	1.78 ± 0.16	0.104
lle	0.95 ± 0.08	1.00 ± 0.06	0.241
Met	0.58 ± 0.04	0.62 ± 0.06	0.116
Thr	0.95 ± 0.06	0.99 ± 0.08	0.313
His	0.98 ± 0.09	1.11 ± 0.16	0.125
Arg	1.34 ± 0.09	1.41 ± 0.11	0.253
Tyr	0.79 ± 0.04	0.83 ± 0.1	0.360
TAA	19.42 ± 1.04	20.55 ± 1.75	0.205
EAA	8.54 ± 0.48	9.08 ± 0.74	0.171
FAA	8.45 ± 0.45	8.81 ± 0.7	0.307
EAA%	43.99 ± 0.54	44.19 ± 0.29	0.463
FAA%	43.50 ± 0.13	42.91 ± 0.57^*^	0.033

Quantitative data are presented as the mean ± SEM. Significance was established using Student's *t*-test. Differences were considered significant at ^*^P < 0.05. TAA, Total amino acid; EAA, Essential amino acid: Thr, Val, Met, Leu, Ile, Phe and Lys; FAA, Flavor amino acids: Asp, Ala, Arg, Asp, Glu and Gly. Thr, Threonine; Val, Valine; Met, Methionine; Ile, Isoleucine; Leucine, Leu; phenylalanine, Phe; lysine, Lys; aspartic acid, Asp; glutamic acid, Glu; glycine, Gly; proline, Ala; tyrosine, Tyr; hydroxyproline, Arg; proline, Pro.

Notably, the content of glycine, a critical flavor-enhancing amino acid, was significantly higher in the Tibetan pigs, measuring 0.95 ± 0.03 g/100 g, in contrast to 0.89 ± 0.05 g/100 g in black pigs (*p* = 0.033), as shown in Table 1. Furthermore, the proportion of flavor amino acids (FAA) relative to total amino acids was also significantly greater in Tibetan pigs at 43.50 ± 0.13%, compared to 42.91 ± 0.57% in black pigs (*p* = 0.033) (Table 1). These findings suggest that Tibetan pigs have a potentially superior amino acid composition and have higher meat sensory quality compared with low-altitude black pigs.

The total amount of LD unsaturated fatty acids in Tibetan pigs and black pigs was similar (Tibetan pigs: 1.13 ± 0.72 g/100 g, black pigs: 1.15 ± 0.57 g/100 g), but there were significant differences in the specific fatty acid composition. Specifically, among the unsaturated fatty acids, α-linolenic acid (C18:3n3; TP: 0.01 ± 0 g/100 g, BP: 0 ± 0 g/100 g; *p* < 0.05), dihomo-γ-linolenic acid (C20:3n6; TP: 0.01 ± 0 g/100 g, BP: 0 ± 0 g/100 g; *p* < 0.05) and arachidonic acid (C20:4n6; TP: 0.04 ± 0.01 g/100 g, BP: 0.02 ± 0.01 g/100 g; *p* < 0.005) of Tibetan pigs were significantly higher than those of black pigs, indicating that Tibetan pigs have an advantage in the accumulation of functional unsaturated fatty acids. In addition, oleic acid (C18:1n9c) is the main component of unsaturated fatty acids in the two pig breeds. The oleic acid content of black pigs (0.87 ± 0.42 g/100 g) is slightly higher than that of Tibetan pigs (0.79 ± 0.56 g/100 g), indicating that black pigs have a slight advantage in the accumulation of monounsaturated fatty acids. Among polyunsaturated fatty acids, linoleic acid (C18:2n6c) has the highest content. The linoleic acid content of Tibetan pigs (0.19 ± 0.09 g/100 g) is slightly higher than that of black pigs (0.15 ± 0.07 g/100 g), further highlighting the characteristics of Tibetan pigs in polyunsaturated fatty acid metabolism (Figure 1F, Supplementary Table S3).

Compared with unsaturated fatty acids, the total amount of saturated fatty acids in black pigs (0.84 ± 0.53 g/100 g) is slightly higher than that of Tibetan pigs (0.71 ± 0.49 g/100 g), showing a significant difference in fatty acid composition between the two pig breeds. Among saturated fatty acids, palmitic acid (C16:0) is the main component, and the palmitic acid content of black pigs (0.49 ± 0.23 g/100 g) is slightly higher than that of Tibetan pigs (0.46 ± 0.31 g/100 g). In addition, the content of stearic acid (C18:0) is high in both pig breeds, but the stearic acid content of black pigs (0.26 ± 0.13 g/100 g) is higher than that of Tibetan pigs (0.23 ± 0.15 g/100 g). These results indicate that black pigs have an advantage in the synthesis or deposition of saturated fatty acids (Figure 1F, Supplementary Table S3).

### 3.3 Metabolome of intestinal contents of Tibetan pigs and black pigs

**Figure 2:**
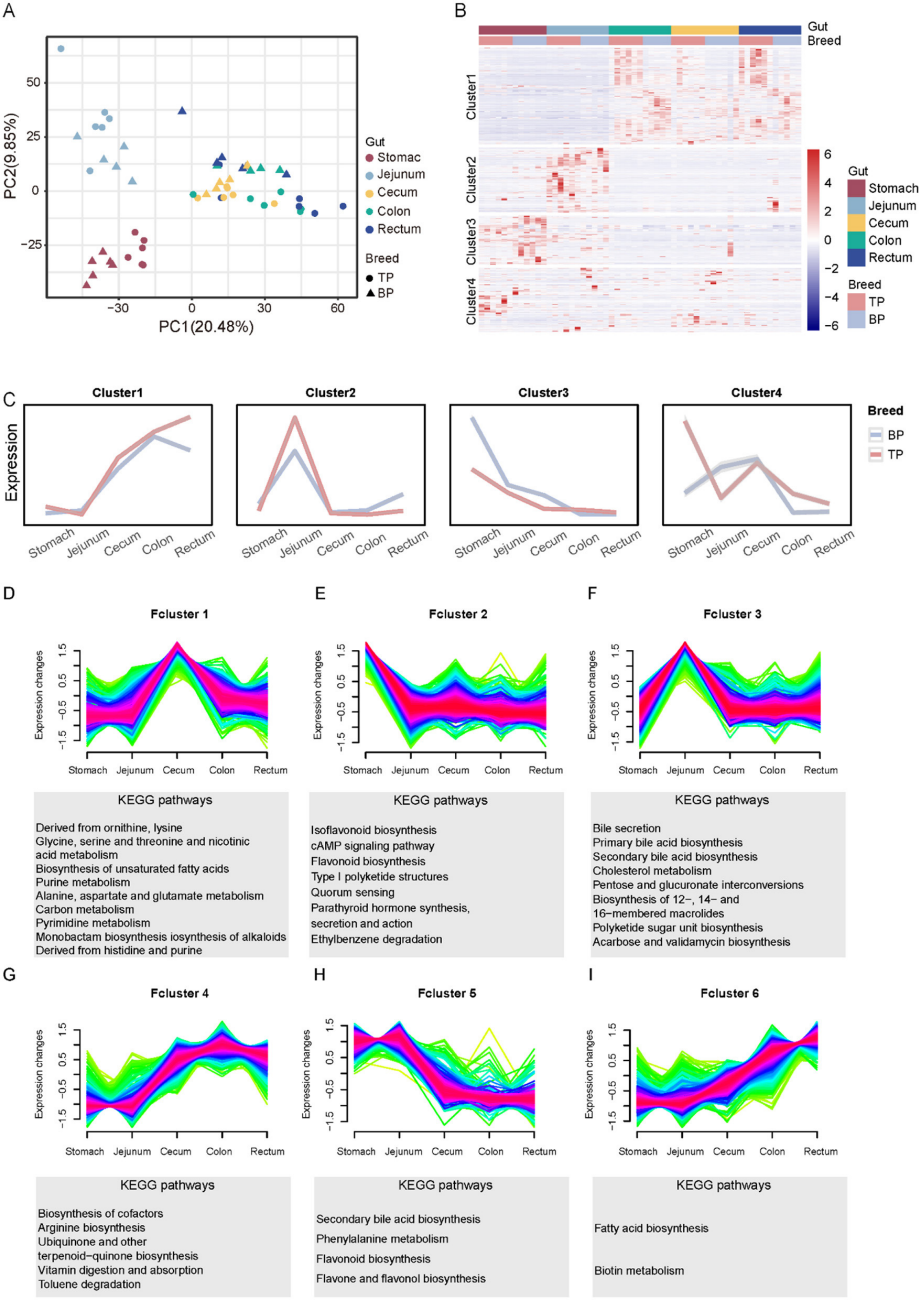
Stratified cluster analysis of metabolites in different gut segments (Stomach, Jejunum, Cecum, Colon, Rectum) of Tibetan pigs and Black pigs. **(A)** PCA analysis of intestinal metabolite contents in different intestinal segments of Tibetan and black pigs; **(B)** Clustered heat map of intestinal metabolites in different intestinal segments of Tibetan pigs and black pigs. **(C)** Four clusters (Clusters 1–4) and trend lines representing changes in intestinal levels of metabolites. **(D–I)** Dynamic expression landscape of gut metabolites in different parts of Tibetan pigs and black pigs. Fuzzy clustering of expression data with six clusters (Fclusters 1–6). Purple and red lines correspond to genes with high membership values. The *y*-axis represents normalized expression values from Mfuzz results.

Different intestinal regions exhibit significant variations in the absorption, transformation, and function of metabolites, with these zonal characteristics determining their unique roles in nutritional metabolism, immune regulation, and environmental adaptation. To investigate the distribution patterns and functional differences of intestinal metabolites in Tibetan pigs and black pigs, this study employed UHPLC-MS/MS technology for a comprehensive metabolomics analysis of intestinal contents. A total of 15,524 peaks were detected, characterized by clear peak shapes and relatively uniform distribution (Supplementary Figure S1, Supplementary Table S4). Database comparison identified 4,737 metabolites, revealing significant differences in metabolite composition between Tibetan pigs and black pigs, as demonstrated by principal component analysis (PCA). Furthermore, PCA indicated that the distribution of metabolites across different intestinal segments exhibited distinct regional characteristics. For instance, the stomach and jejunum displayed relatively unique metabolite compositions, while the, cecum, colon, and rectum, shared a more similar metabolite profile (Figure 2A). Hierarchical clustering analysis further subdivided the metabolites classified above level 4 (Supplementary Table S4) into four clusters based on their change patterns (Figure 2B). The relative concentrations of metabolites in cluster 1 were higher in the large intestine (cecum, colon, and rectum) compared to the stomach and jejunum, with higher levels observed in Tibetan pigs than in black pigs. Metabolites in cluster 2 were more abundant in the ileum. In contrast, the content of metabolites in clusters 3 and 4 was higher in the stomach (Figure 2C).

To further investigate the dynamic expression patterns of metabolites, we employed fuzzy clustering analysis, which categorized the metabolites into six functional clusters (Fcluster 1–6) as illustrated in Figures 2D–I. In the stomach, metabolites from Fcluster 2 predominantly partake in the secretion of digestive enzymes and hormones, and the transmission and activation of various cellular signals, underscoring the stomach's pivotal role in preliminary food digestion and digestive system regulation. In the jejunum, metabolites from Fcluster 3, including bile acids and cholesterol, facilitate the digestion and absorption of fats and fat-soluble vitamins, as well as the comprehensive metabolism of cholesterol, thereby reflecting the jejunum's central function in lipid metabolism and energy absorption. The cecum utilizes metabolites from Fcluster 1 to activate pathways related to amino acid and carbon metabolism, demonstrating its essential role in the further breakdown and absorption of nutrients. Meanwhile, in the colon, Fcluster 4 metabolites primarily engage in arginine biosynthesis and cofactor biosynthesis, aiding in protein synthesis and the digestion and absorption of vitamins. Metabolites in Fcluster 6 from the rectum are chiefly involved in fatty acid biosynthesis and biotin metabolism, emphasizing their contribution to energy storage and structural maintenance. These metabolites' region-specific expression patterns not only reflect the intestinal division of labor in digestion, absorption, and metabolism but also highlight its crucial role in adapting to various nutritional states. The comprehensive hierarchical and fuzzy clustering analyses reveal that the distribution of intestinal metabolites in Tibetan and black pigs exhibits significant regional and functional specificity.

### 3.4 Analysis of differential metabolites and their correlation between the intestines of BP pigs and TP pigs

**Figure 3:**
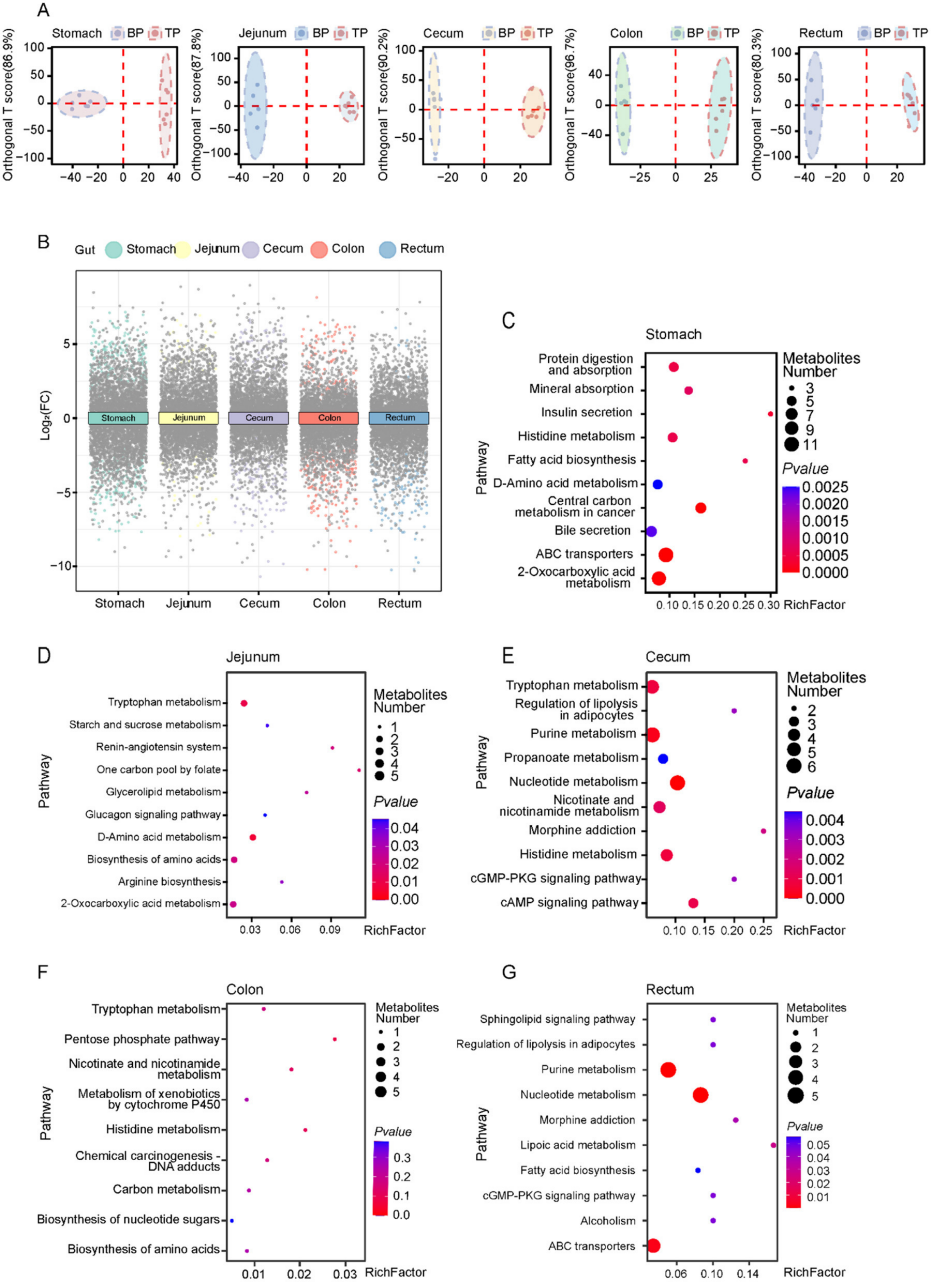
Differential metabolite analysis between Tibetan pigs and black pigs. **(A)** Orthogonal partial least squares discriminant analysis (OPLS-DA) analysis of metabolites in Tibetan pigs and black pigs; **(B)** Multiple volcano plots showing DEMs in TP and BP groups in different intestinal segments; **(C–G)** KEGG pathway enrichment analysis of DEMs in various intestinal sites between Tibetan pigs and Black pigs groups.

To explore how Tibetan pigs and black pigs optimize energy utilization and adapt to hypoxic stress through intestinal metabolic regulation, we performed OPLS-DA and differential fold analysis to identify differential metabolites (Figures 3A, B, Supplementary Figures S2, S3). We identified a total of 900 differential metabolites across different intestinal segments, with 367 metabolites upregulated and 533 metabolites downregulated (Supplementary Table S5). Through KEGG enrichment analysis, we clarified the specific biological pathways associated with the differential metabolites and identified 78 key pathways (*p* < 0.05). Among them, the differential metabolites in the stomach were mainly enriched in protein digestion and absorption, fatty acid synthesis, and mineral absorption pathways, which support energy storage and oxygen transport in cold environments (Figure 3C). In the jejunum and colon, the significantly enriched tryptophan metabolism and glycolysis pathways help regulate serotonin levels and rapid energy supply to adapt to low oxygen pressure (Figures 3D–F). The differential metabolites in the cecum are concentrated in the purine metabolism and fatty acid synthesis pathways, which play a key role in nucleic acid repair and energy storage (Figure 3E). The metabolites in the rectum are enriched in the fatty acid biosynthesis and ABC transporter pathways, supporting nutrient transport and energy storage (Figure 3G). Compared with black pigs, Tibetan pigs show stronger metabolic adaptability to the extreme environment of the plateau, especially in energy metabolism and oxygen utilization efficiency. In particular, the metabolites of Tibetan pigs in the stomach and cecum are significantly enhanced in the protein digestion and absorption and fatty acid synthesis pathways. In addition, the enhanced purine metabolism in the cecum suggests that Tibetan pigs may have more efficient nucleic acid repair mechanisms, which is particularly critical for the protection and repair of DNA in harsh environments.

**Figure 4:**
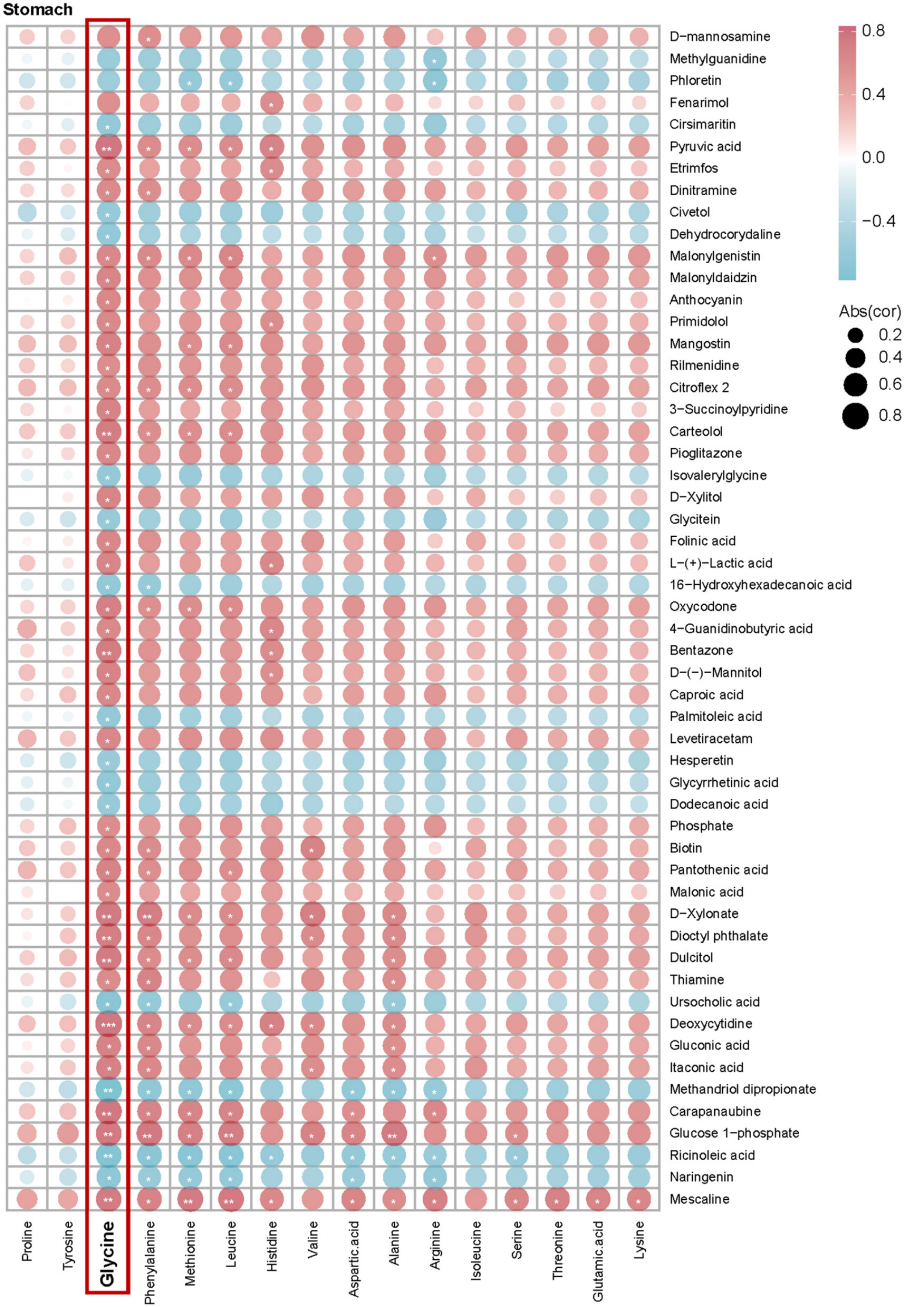
Correlation analysis between intestinal differential metabolites and muscle amino acid content between Tibetan pigs and black pigs. Spearman correlation coefficient values showing the relationship between DEMs in the stomach and muscle amino acid indices. Red and blue colors indicate positive and negative correlations, respectively, between each measurement and the species shown.

**Figure 5:**
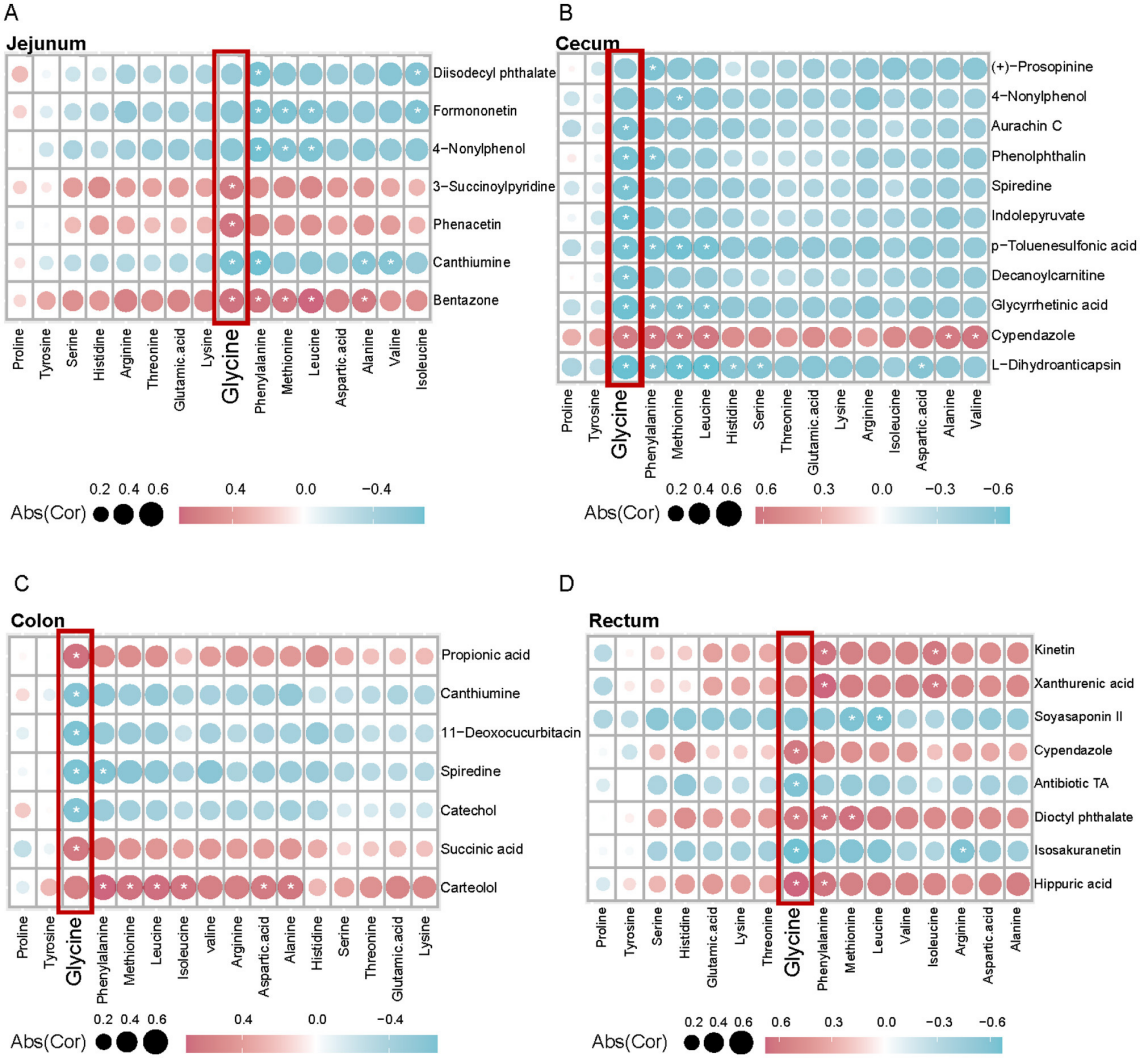
Correlation analysis between intestinal differential metabolites and muscle amino acid content between Tibetan pigs and black pigs. **(A–D)** Spearman correlation coefficient values showing the relationship between DEMs in (**A**, Jejunum; **B**, Cecum; **C**, Colon; **D**, Rectum) and muscle amino acid indicators in different intestinal sites. Red and blue indicate positive and negative correlations between each measurement and the species shown, respectively.

Muscle tissue is critical for adaptation to high altitudes, requiring increased amino acids in hypoxic environments to maintain metabolic equilibrium and respond to shifts in energy requirements. In examining the interactions between glycine and intestinal metabolites and their impact on muscle phenotype, we observed differential associations of glycine with DEMs across various intestinal sites (stomach, jejunum, cecum, colon, and rectum), as well as significant correlations with muscle amino acid indicators (Figures 4, 5A–D). In the stomach, a majority of DEMs exhibited strong positive correlations with glycine, indicating that metabolites expressed by the gastric microbiome are intricately linked to muscle glycine levels and that gastric metabolic processes significantly influence muscle glycine content (Figure 4). Conversely, in the cecum, the observed negative correlations between DEMs and glycine suggest differing patterns of microbial metabolic regulation (Figure 5B). Furthermore, the levels of ricinoleic acid, linoleamide, niacin, and linoleic acid in the intestine were positively correlated with the levels of linolenic acid, eicosatrienoic acid, eicosadienoic acid, and oleic acid in the muscle. Conversely, the levels of docosahexaenoic acid, tetracosahexenoic acid, and chenodeoxycholic acid in the intestine were negatively correlated with most fatty acids in the muscle (Supplementary Figure S4). These findings suggest that glycine acid and linoleic acid levels in the intestine may serve as indicators of high-quality amino acids and fatty acids.

### 3.5 Identification and functional enrichment of differentially expressed genes (DEGs)

**Figure 6:**
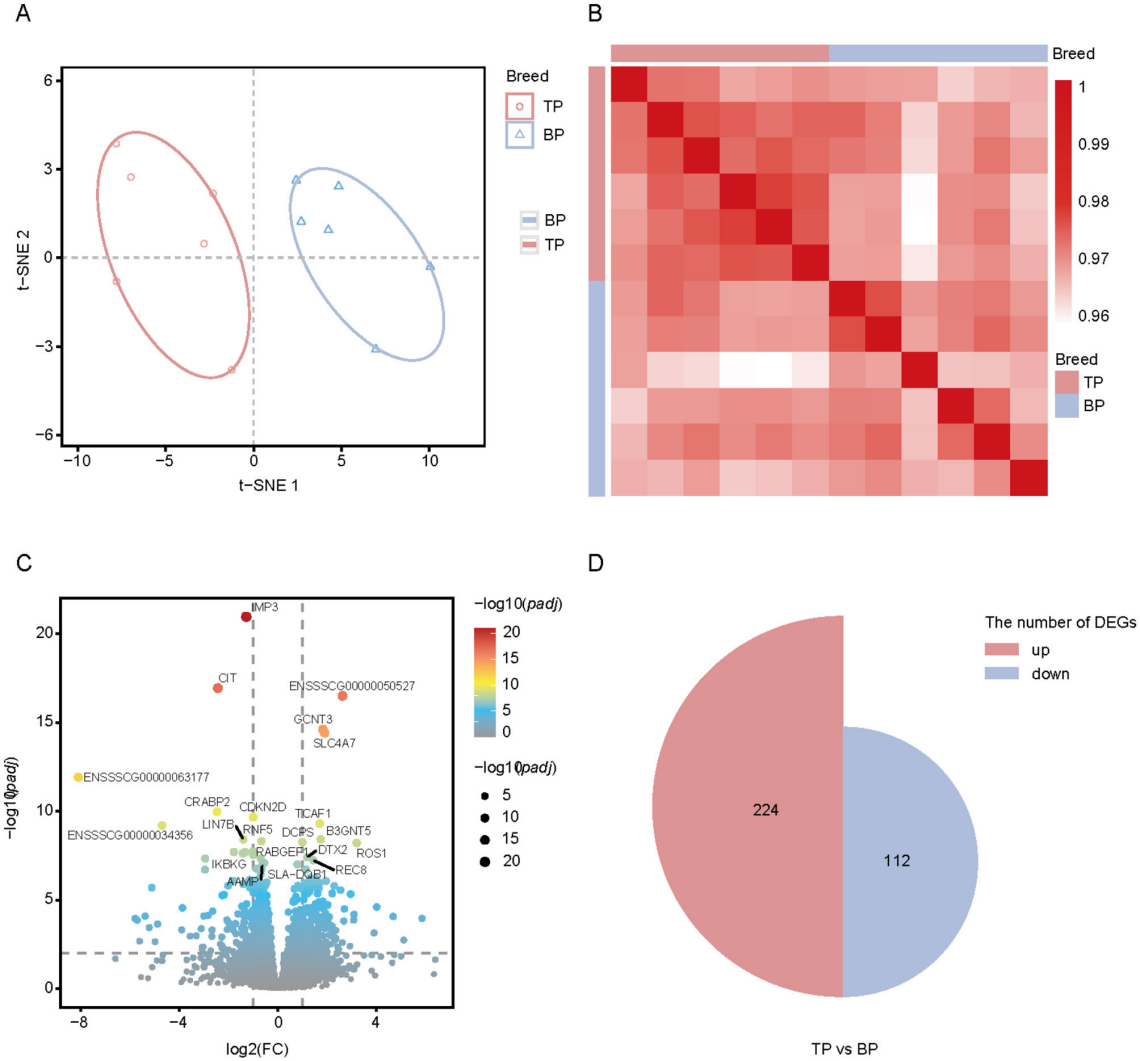
Transcriptome analysis of Tibetan pig and black pig samples. **(A)** t-SNE analysis of Tibetan pig and black pig samples. First and second dimensions are shown; **(B)** Spearman correlation heatmap showing the correlation between each RNA-seq sample. Color spectrum ranges from white, which indicates a low correlation, to red, which indicates a high correlation; **(C)** Volcano map showing the differentially expressed genes of the *LD* muscle in TB and BP groups. Red (upregulated genes) and blue (downregulated genes) dots represent differentially expression genes. Gray dots represent similarly expressed genes; **(D)** Proportional area plot (semicircle). Two groups of semicircles represent DEGs in the two groups of muscles, and the area represents the number of DEGs.

We analyzed RNA-seq data from 12 samples of Tibetan pigs and black pigs LD. We generated 280 million paired-end reads (2 × 150 bp), totaling 85.4 Gb of clean data. The average percentage of clean reads that mapped to the pig reference genome (Sus scrofa 11.1) was 98.46%, with a range of 98.22%−98.78% (Supplementary Table S6). t-SNE analysis revealed that the 12 samples clustered distinctly according to breed, with Tibetan pigs and black pigs separated along t-SNE1 (Figure 6A). The highest correlation was observed among the six replicate samples from the same breed (Figure 6B). We conducted differential expression analysis to identify genes with significant expression differences between the two breeds, using *p*_adj_ < 0.01 and |log_2_foldchange| > 2 as criteria (Figure 6C). A total of 336 DEGs were identified between the TP and BP groups, with 224 genes upregulated in the TP group and 112 genes upregulated in the BP group (Figure 6D).

**Figure 7:**
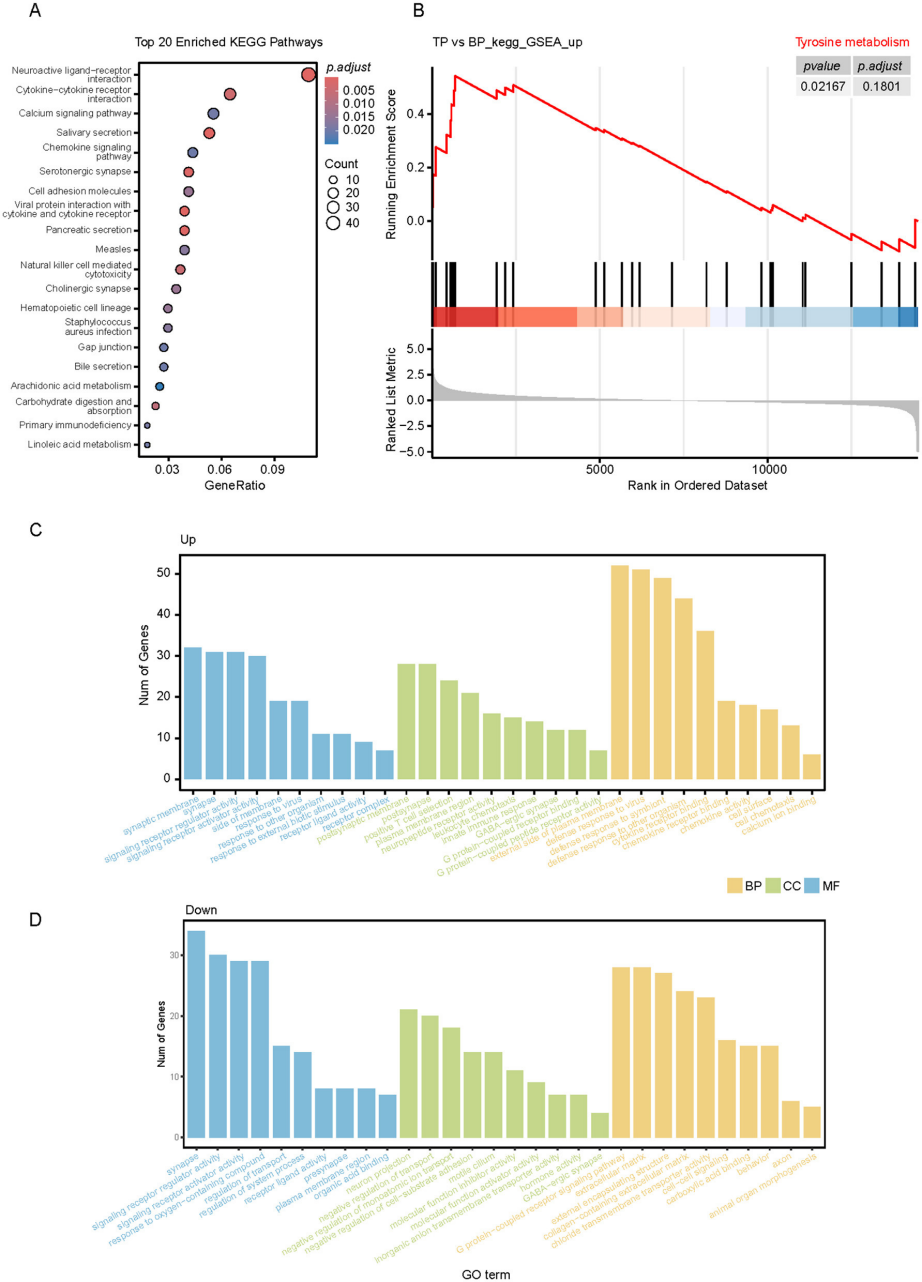
Functional annotation analysis of DEGs in muscles of TP and BP groups. **(A)** KEGG pathway enrichment analysis of muscle DEGs in TP and BP groups; **(B)** GSEA plot showing the enrichment analysis of tyrosine metabolism between joint BP and TP muscles. The table in the upper right corner shows the *p*_adj_ values for each term. **(C, D)** Gene ontology (GO) enrichment analysis of DEGs between TP **(C)** and BP **(D)** muscles.

To investigate the functions of DEGs between TP and BP tissues, we performed KEGG and GO functional enrichment analyses. The KEGG analysis revealed significant enrichment of these genes in several metabolic pathways, including salivary secretion, pancreatic secretion, carbohydrate digestion and absorption, linoleic acid metabolism, bile secretion, and arachidonic acid metabolism (Figure 7A). In muscle tissues, the levels of amino acids and most fatty acids were higher in the LD of BP compared to TP (Figures 1D, E). To validate this observation and identify key pathways affecting muscle meat quality, we conducted Gene Set Enrichment Analysis (GSEA) on the DEGs, focusing on metabolism-related genes. In the TP group, most genes in the tyrosine metabolic pathway were significantly upregulated (Figure 7B). In the BP group, gene expression in the adipocytokine signaling pathway was significantly upregulated (Supplementary Figure S5B). Additionally, GO enrichment analysis showed that the DEGs in both groups were primarily involved in biological regulation, metabolic processes, and cellular processes (Figures 7C, D).

**Figure 8:**
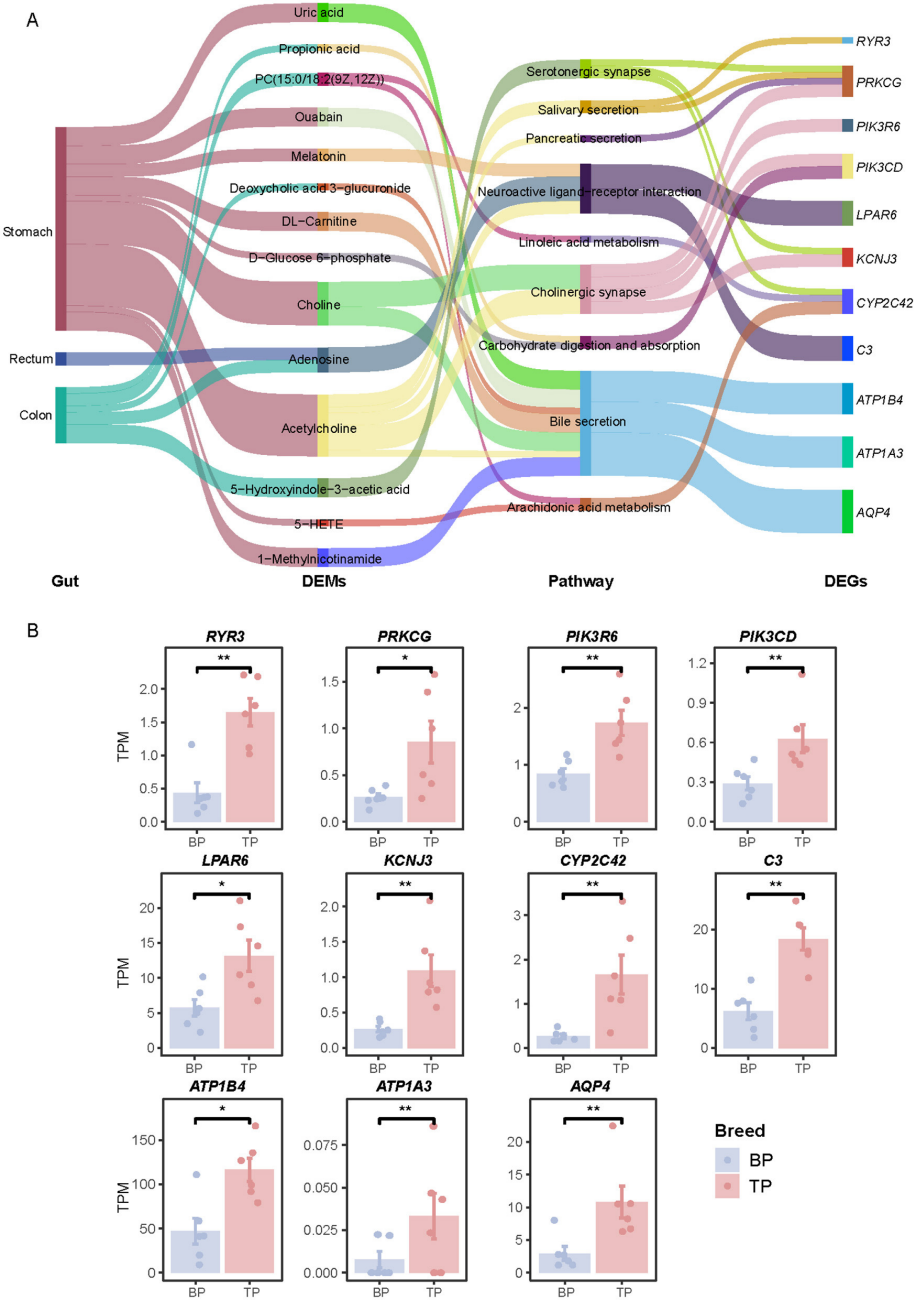
Transcriptome-metabolome association analysis. **(A)** Correlation network between DEGs and DEMs between TP and BP muscles. **(B)** Histograms representing the relative expression levels of DEGs in TPM of two pig breeds. Differences were considered significant at *P < 0.05, **P < 0.01.

This study integrated transcriptomic and metabolomic data and systematically analyzed the interactions between the muscle transcriptome and metabolites in different intestinal parts to comprehensively reveal the underlying molecular mechanisms driving phenotypic differences. Specifically, we enriched DEGs and DEMs in different intestinal parts into corresponding KEGG pathways, respectively, and identified common enriched pathways. Further investigation into the relationship between DEGs and DEMs across different intestinal segments was conducted by calculating the Pearson correlation coefficient, with a correlation threshold set at |CC| > 0.7 and a significance level of *p* < 0.01. Our findings identified nine critical KEGG pathways involving 11 genes (*RYR3, PRKCG, PIK3R6, PIK3CD, LPAR6, KCNJ3, CYP2C42, C3, ATP1B4, ATP1A3*, and *AQP4*). These genes are significantly associated with 14 key metabolites, including propionate, deoxycholic acid-3-glucoside, and adenosine. These associations predominantly occur through pathways critical for bile secretion, carbohydrate metabolism, and neurotransmitter regulation, as shown in Figure 8A. The main enriched area of differential pathways is the stomach, where most metabolites are related to bile acid secretion and regulate the expression of acetylcholine and *ATP1B4, ATP1A3*, and *AQP4* genes through this pathway. Comparative analysis showed that the expression levels of acetylcholine, *RYR3, PIK3CD, ATP1A3, AQP4*, and other genes were significantly higher in plateau Tibetan pigs than in plain Tibetan pigs, as illustrated in Figure 8B and Supplementary Figure S6A. Additionally, the protein encoded by the *ATP1A4* gene, a member of the Na^+^/K^+^-ATPase subfamily (28), plays a crucial role in establishing and maintaining the electrochemical gradients of sodium and potassium ions across cell membranes. These gradients are vital for regulating osmotic pressure, sodium-coupled transport of various molecules, and the electrical excitability of nerves and muscles (29).

## 4 Discussion

Pork is a key source of high-quality protein in human nutrition, providing a rich profile of essential amino acids critical for health (30, 31). The protein quality of pork, and its overall nutritional value, largely depend on its amino acid composition (32). In this study, the unique muscle fiber characteristics of Tibetan pigs, including smaller fiber areas and higher oxidation levels, along with an increased content of flavor-enhancing amino acids, underscore their suitability for high-quality meat production (33–35). Long-chain n-3 polyunsaturated fatty acids (LC-PUFA) are vital for maintaining immune function (36), promoting skin and hair health (37), and reducing the risk of chronic diseases (38, 39). Furthermore, maintaining an optimal dietary balance between n-3 and n-6 fatty acids is crucial (40). A high n-6/n-3 ratio may enhance inflammatory responses and increase the risk of chronic diseases 37 (41). Our results indicated that while the n-3 fatty acid content did not significantly differ between BP and TP pigs, the n-6/n-3 ratio was notably lower in BP pigs (~42.1%), suggesting that the fatty acid composition in BP pigs may be more beneficial in reducing inflammation and the associated risk of chronic diseases. Notably, the arachidonic acid content in TP pigs was significantly higher than in BP pigs. Arachidonic acid, a polyunsaturated n-6 fatty acid, can be converted into pro-inflammatory prostaglandins and leukotrienes (42), which play important roles in regulating immune responses and inflammatory processes.

The digestive tracts of Tibetan pigs and their counterparts, such as black pigs, exhibit adaptations to their specific diets and environmental conditions (23, 43). The differences in metabolite profiles across various sections of their digestive tract (stomach, jejunum, cecum, colon, rectum) demonstrate evolutionary strategies geared toward optimizing digestive efficiency and nutrient absorption under distinct ecological pressures. Notably, the stomach and jejunum of Tibetan pigs show enhanced pathways for protein digestion, absorption, and fatty acid synthesis—crucial adaptations for survival in high-altitude areas where energy requirements are high, yet oxygen supply is compromised. Enhanced protein digestion is vital for maintaining muscle mass and overall physiological resilience, while increased fatty acid synthesis provides essential energy reserves needed to combat the cold (44, 45). In contrast, pig breeds adapted to more temperate or low-altitude environments often exhibit boosted carbohydrate metabolism to quickly meet energy needs (6). For example, commercial breeds like Yorkshire pigs display enhanced carbohydrate and lipid metabolism, suited to their energy-dense diets and less stressful environments (6, 7). The metabolic specializations observed in the cecum and rectum of Tibetan pigs, particularly in purine metabolism and fatty acid synthesis pathways, suggest adaptations for efficient nucleic acid repair and sustained energy storage, likely a response to the cellular renewal demands and damage repair necessitated by UV exposure and hypoxic stress at high altitudes (46). A diet rich in proteins and fats is thus ideal for Tibetan pigs, supporting their unique metabolic pathways and enhancing their adaptability and survival in severe conditions (47). This metabolic insight also lays the groundwork for diet optimization in other species or breeds, aiming to develop precise nutritional strategies tailored to their specific environmental and physiological challenges, ultimately enhancing health and productivity.

To identify potential biomarkers for meat quality, we performed a correlation analysis between DEMs and associated phenotypic traits. Our results show that the concentrations of tryptamine, 6-methylquinoline, and lactic acid in the intestine are directly correlated with the levels of phenylalanine, glycine, leucine, and histidine in muscle tissue. Extensive research supports the role of intestinal lactic acid bacteria in modulating and enhancing meat quality and flavor (48–50), which aligns with our findings. We also observed a negative correlation between muscle amino acids and certain metabolites in the intestine, such as L-tyrosine, ricinoleic acid, stearoylglycerol, pyroglutamic acid, and arachidonic acid. However, there is currently a lack of empirical studies directly examining the impact of intestinal fatty acids on skeletal muscle protein metabolism. This phenomenon warrants further investigation. Additionally, we found a positive correlation between intestinal linoleic acid and muscle fatty acids, including linolenic acid, eicosatrienoic acid, and oleic acid. The composition of meat fatty acids can be influenced by diet, which is more easily manipulated in monogastric animals such as pigs and poultry (51). By adjusting dietary linoleic acid levels, we can effectively control the n-6 to n-3 polyunsaturated fatty acid ratio in meat products (52). This approach provides a feasible strategy to improve the nutritional value of meat from monogastric livestock. Among the differential metabolites identified in the intestine, linoleic acid was positively correlated with fatty acid content. Biochemical pathways convert linoleic acid to arachidonic acid through the action of fatty acid desaturase and elongase enzymes (53). Arachidonic acid is then metabolized by cyclooxygenase and lipoxygenase into key inflammatory mediators, including prostaglandins (e.g., PGE2) and leukotrienes (e.g., LTB4) (42). These mediators are critical in regulating inflammatory responses and the oxidative metabolism of fatty acids in muscle cells, influencing energy mobilization and storage (54). This association suggests that linoleic acid levels in the gut could be indicative of muscle mass.

In a comprehensive transcriptomic analysis, we identified 336 DEGs between tissue phenotypes TP and BP, highlighting genetic variations that may explain differences in muscle quality. These DEGs were notably enriched in metabolic pathways essential for nutrient digestion, absorption, and metabolism. Specifically, pathways involved in salivary and pancreatic secretion, carbohydrate digestion and absorption, linoleic and arachidonic acid metabolism, and bile secretion showed significant enrichment.

In our study, we employed multi-omics techniques to perform a correlation analysis of co-enriched pathways between differential metabolites in the digestive tract and differential genes in muscle tissue. This approach helped us unravel the regulatory network of the digestive tract-muscle axis, specifically focusing on how digestive tract metabolism, muscle gene expression, and signaling pathways are interconnected. Our analysis revealed that differential metabolites located in the stomach, rectum, and colon show significant correlations with muscle gene expression. Notably, the stomach emerged as the primary site for these correlated metabolites, indicating its pivotal role in influencing muscle physiology (55). This may be attributed to several factors: (1) Food undergoes initial digestion in the mouth and esophagus but is not absorbed at these stages. (2) The stomach serves as the main organ for food storage and digestion (56), where mechanical and chemical processes release a substantial amount of metabolites (57). Despite its role in digestion, the stomach has a relatively low nutrient absorption capacity, primarily absorbing only small quantities of substances like alcohol, some water, and inorganic salts. (3) The small intestine is responsible for absorbing about 90% of nutrients (58), resulting in a lower concentration of metabolites in other parts of the digestive tract compared to the stomach. Among the metabolites analyzed, acetylcholine was found to have a significant correlation with muscle activity. It is well-documented that motor neurons release acetylcholine at the neuromuscular junction, influencing muscle contraction by acting on muscle cell receptors. Our findings suggest that dietary acetylcholine could enter the body and affect muscle tissue, highlighting an exogenous pathway for muscle modulation. Further pathway enrichment analysis indicated that acetylcholine primarily influences muscle contraction through mechanisms like cholinergic synapses and neuroactive ligand-receptor interactions. Additionally, there is a minor impact on the secretion processes of various digestive glands, including those producing saliva, pancreatic enzymes, and bile.

A significant discovery from our research is the identification of bile secretion as a primary signaling pathway that links metabolites in the digestive tract to muscle function (59, 60). This connection is predominantly mediated by the differential expression of three specific muscle genes: *ATP1B4, ATP1A3*, and *AQP4* in muscle tissues. Notably, the ATP1B4 gene encodes BetaM-proteins, which are the sole proteins specific to skeletal and atrial cardiac muscles of the inner nuclear membrane found in eutherian mammals (61, 62). In murine models, the absence of BetaM, resulting from an *Atp1b4* gene knockout, leads to a marked decrease in body size and weight, developmental delays, and elevated neonatal mortality rates (62). Transcriptomic analysis via mRNA sequencing of skeletal muscle from both neonatal wild-type and *Atp1b4* knockout mice demonstrated a significant down-regulation of fast-twitch muscle genes alongside an up-regulation of slow-twitch muscle genes (63). This was accompanied by widespread changes in the expression of genes that regulate lipid metabolism. In stark contrast, single-cell sequencing data from human skeletal muscle tissues showed an extremely high positive correlation between *ATP1B4* and markers of oxidative muscle fibers, namely TNNI1 (0.9976) and MYH2 (0.9917) (64). Our investigations into pigs, particularly Tibetan pigs known for their robust oxidative muscle fibers, revealed a higher expression of the *ATP1B4* gene, similar to findings in humans. Given these consistent observations across species, it appears that the *ATP1B4* gene's function in pig muscle mirrors that in human muscle. This similarity suggests a promising avenue for further research, specifically the development of an *ATP1B4* gene knockout pig model. Such a model would provide valuable insights into the gene's role in muscle physiology, potentially influencing nutritional strategies and management practices for farm animals and offering implications for human health research (65).

## 5 Conclusions

This comprehensive metabolomic analysis of intestinal sections from TP and Sichuan BP reveals that BP pigs demonstrate superior meat quality compared to TP pigs. We identified key metabolites, including linoleic acid and lactic acid, that positively correlate with favorable meat quality parameters, such as amino acid profiles and fatty acid ratios in muscle. These metabolites suggest the potential for enhancing meat quality through dietary adjustments, as modifications in feed composition can effectively alter the intestinal metabolite landscape. This study provides a strong theoretical foundation for using precision agriculture techniques to improve meat quality in specific pig breeds.

## Data Availability

The RNA-Seq data of skeletal muscle of Tibetan pigs and black pigs have been deposited in the National Center for Biotechnology Information (NCBI) Sequence Read Archive (SRA) database, with the BioProject accession number PRJNA1192560.
