# Supplementary material for: Insights into high-altitude adaptation and meat quality regulation by gastrointestinal metabolites in Tibetan and black pigs
Source: Front Vet Sci. 2025 Mar 26;12:1569196. doi: 10.3389/fvets.2025.1569196 (PMC11979216; doi:10.3389/fvets.2025.1569196)
Supplement: Supplementary file 1 [file Table_1.docx]

Supplementary Material

**Insights into High-Altitude Adaptation and Meat Quality Regulation by Gastrointestinal Metabolites in Tibetan and Black Pigs**
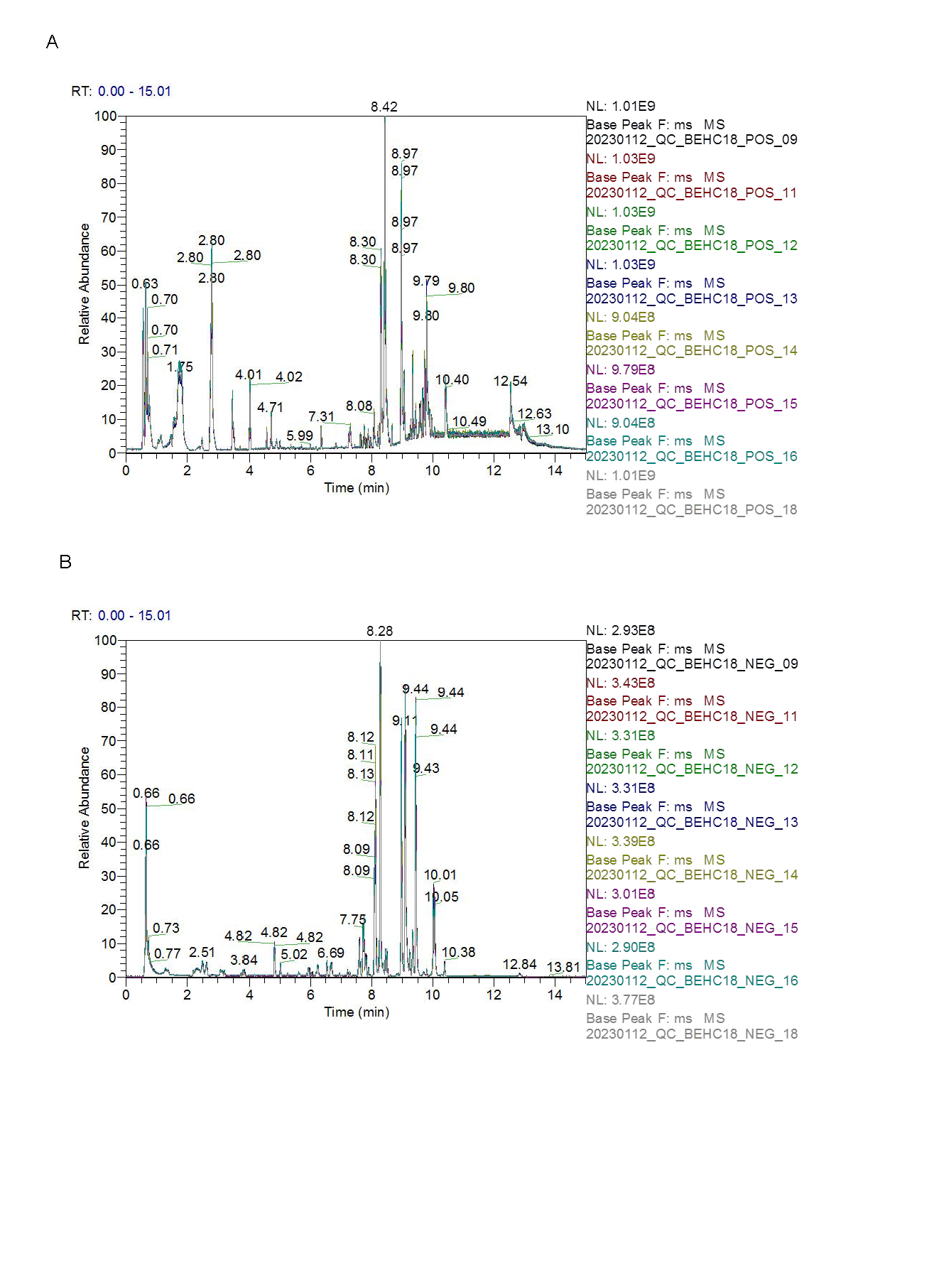


**Figure S1.** Total-ion chromatograms of serum metabolites. (A) Positive ion modes; (B) negative ion modes.


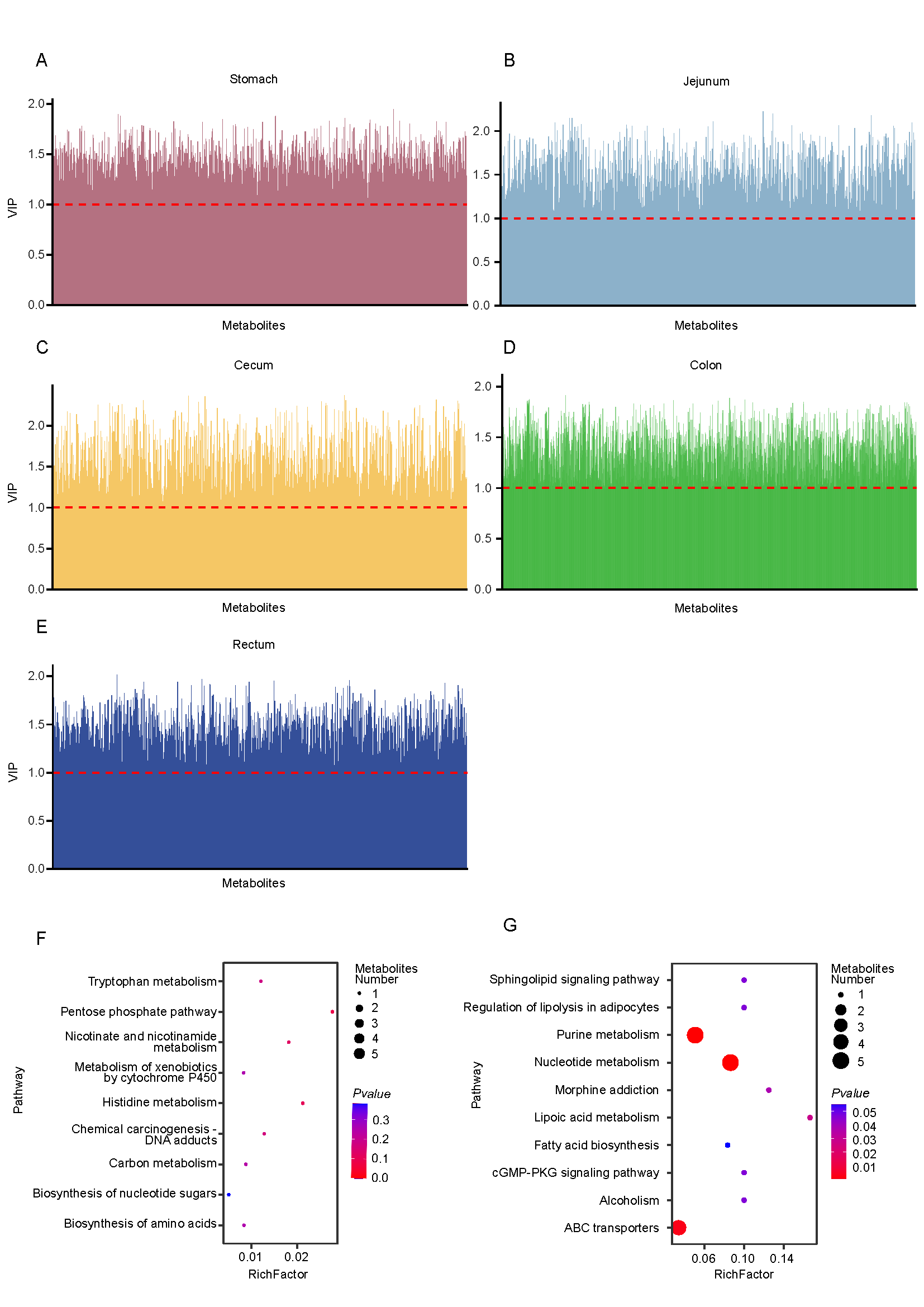


**Figure S2.** (A-E) OPLS-DA (Orthogonal Partial Least Squares Discriminant Analysis) analysis of VIP scores of Tibetan pig and black pig metabolites


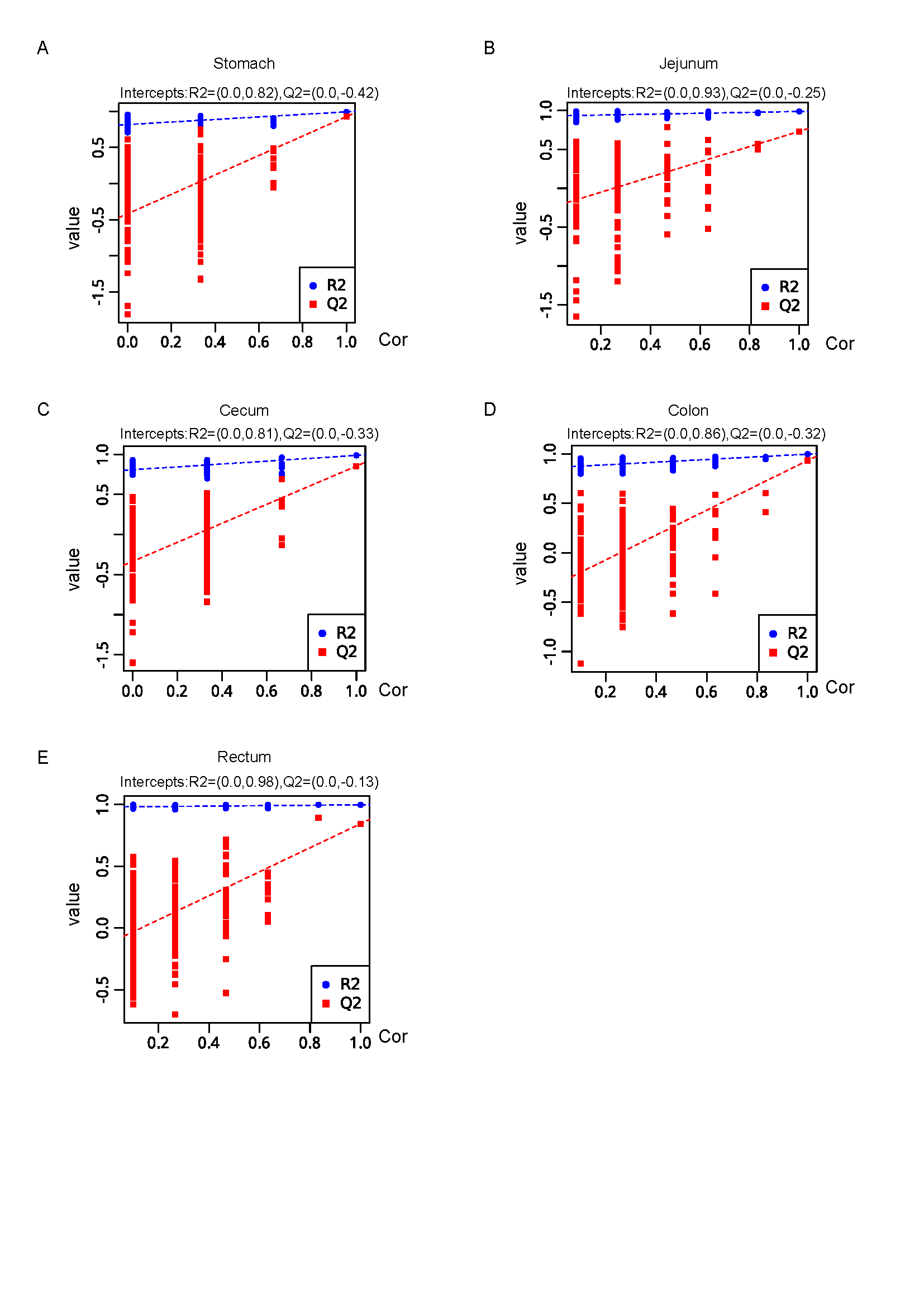


**Figure S3.** Analysis of differential metabolites between Tibetan pigs and black pigs. (A-E) OPLS-DA model was subjected to 200 response permutation tests (RPT, response permutation testing).


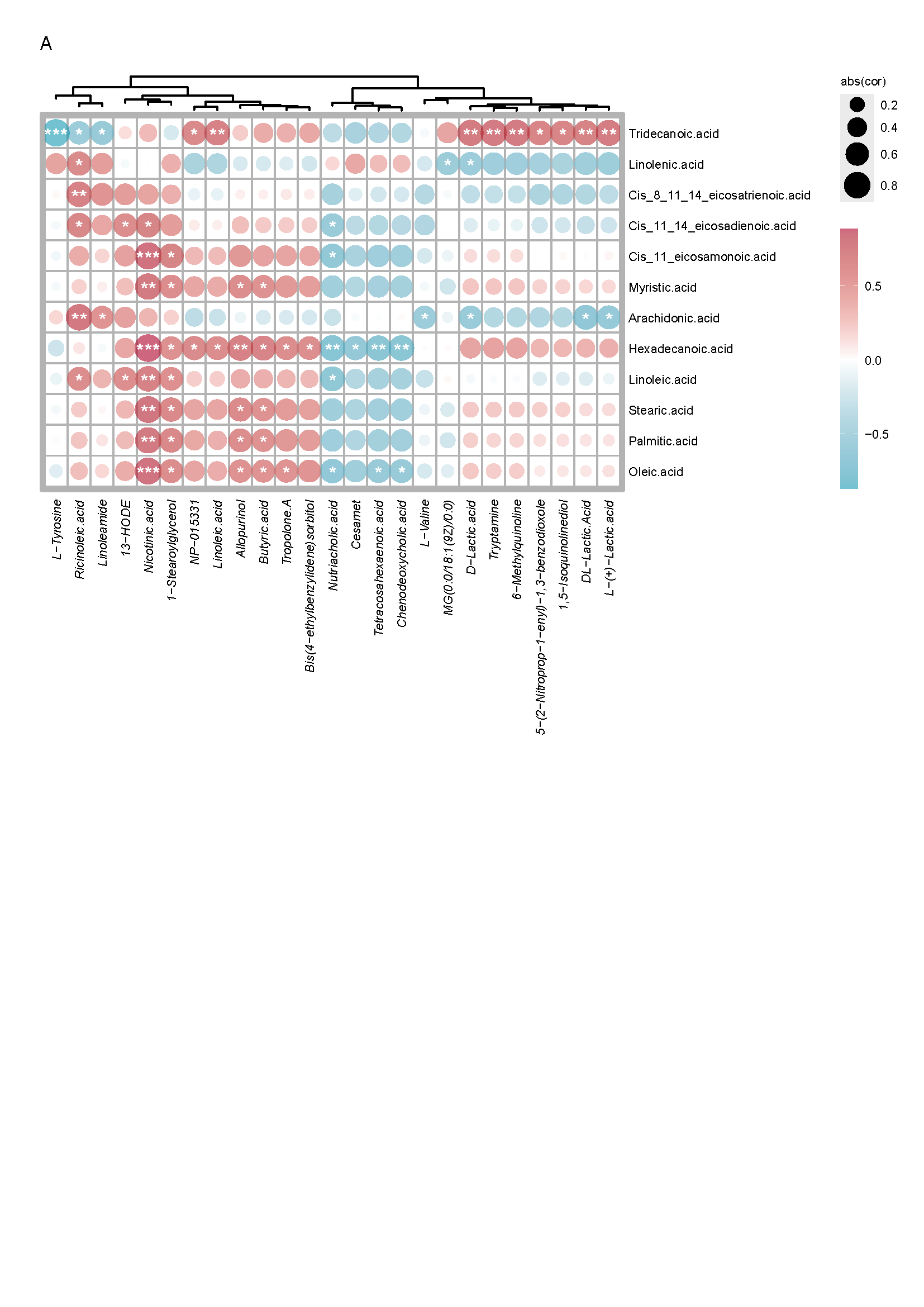


**Figure S4** Spearman correlation coefficient values show the relationship between DEM and muscle fatty acid indices. Red and blue colors indicate positive and negative correlations, respectively, between each measurement and the species shown.


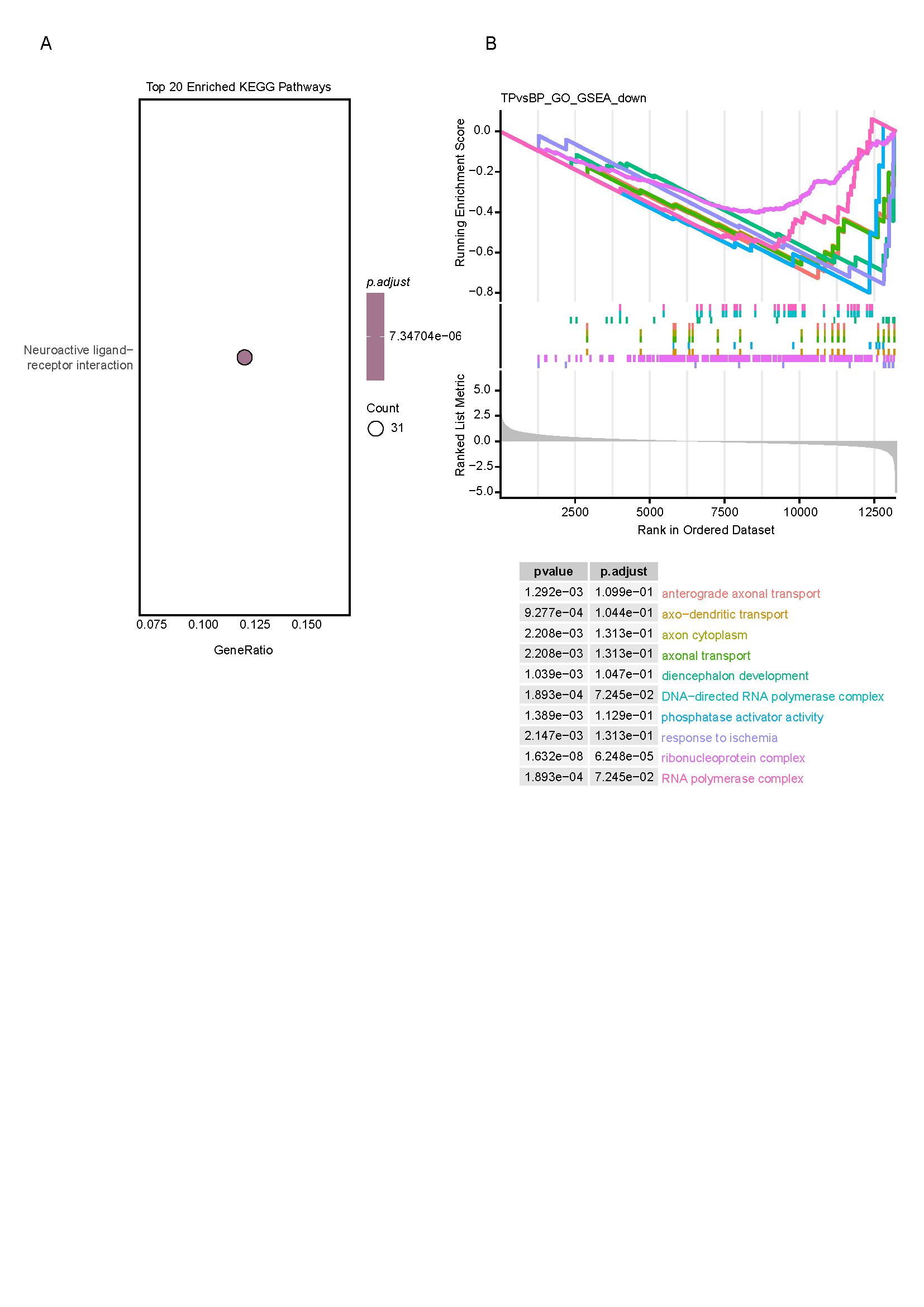


**Figure S5.** (A) KEGG pathway enrichment analysis of muscle DEGs in TP and BP groups; (B) GSEA plot showing top 10 enrichment analysis between joint BP and TP muscles.


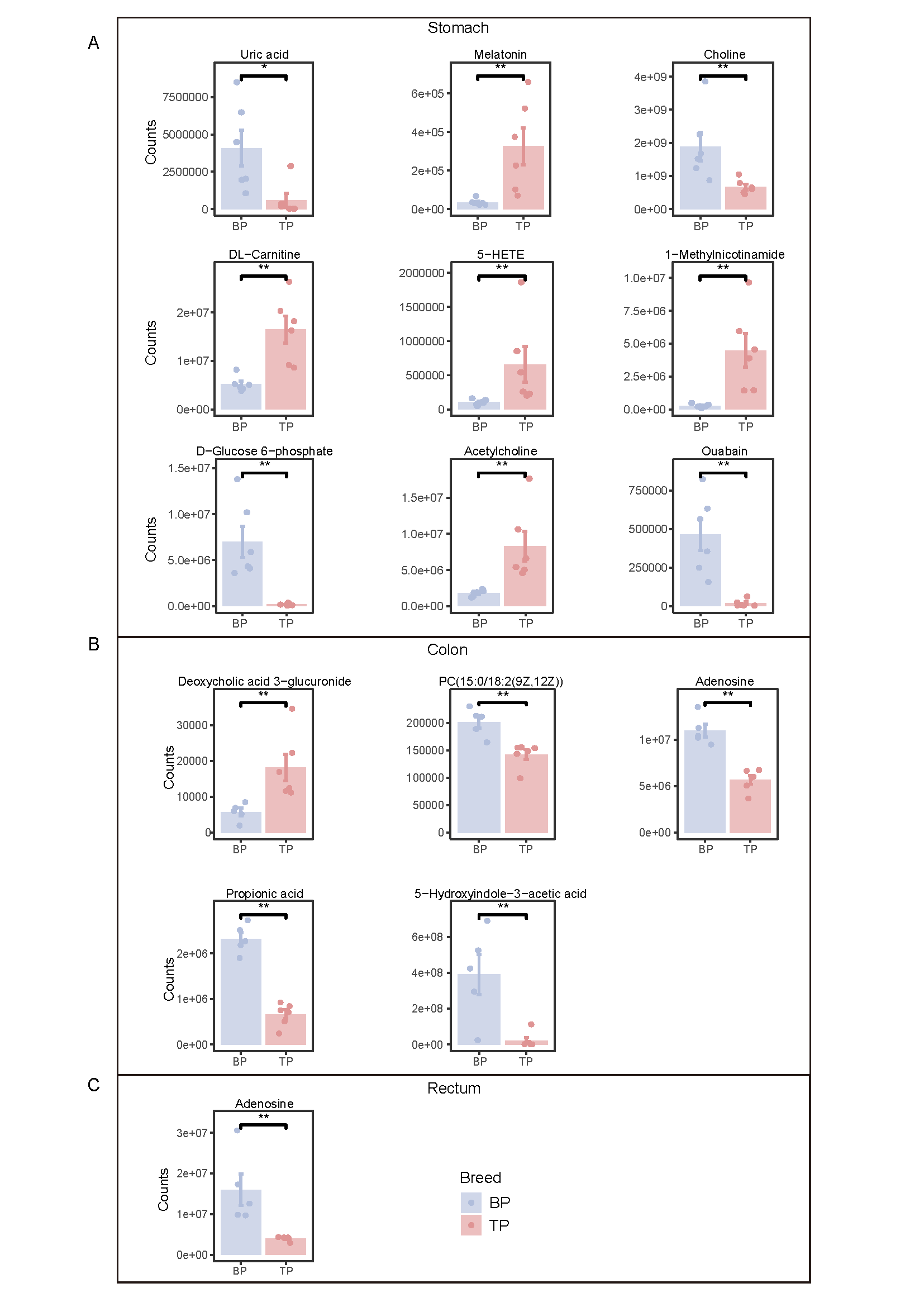


**Figure S6.** (A-C) Bar graph showing the DEMs content of Tibetan pigs and black pigs. Differences were considered significant at **P* < 0.05, ***P* < 0.01 and ****P* < 0.001.

**Supplementary information on animal management and feeding conditions**

During the 300-day stable feeding period, all Tibetan pigs and black pigs were raised under standardized conditions. The following are the details:

1. Feeding environment

Each pig was provided with a feeding space of 3.0 m²/head to ensure sufficient room for activities.

Temperature control: Black pigs were raised at low altitudes (461 m above sea level), with a naturally changing ambient temperature ranging from 15-25°C, and equipped with sunshade and ventilation equipment to cope with hot weather; Tibetan pigs were raised at high altitudes (3,750 m above sea level), with an ambient temperature of 5-15°C, and provided with insulation facilities to ensure the comfort of pigs in cold weather.

Bedding: Straw was provided as bedding during feeding and was changed regularly to keep the pens clean and dry.

2. Diet composition

Feeding a uniformly formulated basic diet, the diet composition is as follows:

Energy feed: corn accounts for about 60%;

Protein feed: soybean meal accounts for about 25%;

Crude fiber feed: bran and silage account for about 10%;

Mineral and vitamin additives: account for about 5%.

Drinking water: All pigs can drink clean water freely, and the drinking water device is cleaned daily to ensure hygiene.

Feeding frequency: Feed twice a day, at 8 am and 5 pm.

3. Feeding management

During the entire feeding cycle, the health status of the experimental pigs was checked regularly to ensure that they were not affected by disease or stress.

No antibiotics or hormone drugs were used for any pigs.
